# Electrospun Quaternary Ammonium Salt-Loaded Nanofibrous Nanocomposites as Wound Dressings

**DOI:** 10.1021/acsomega.6c00475

**Published:** 2026-04-03

**Authors:** Sena Özdil Şener, Sema Samatya Yilmaz, Tahsin Ertaş, Merve Dandan Doganci, Bircan Dinç, Erdinc Doganci

**Affiliations:** † 52980 Kocaeli University, Science Institute, Department of Biomedical Engineering, Kocaeli 41001, Türkiye; ‡ 52980 Kocaeli University, Engineering Faculty, Department of Chemical Engineering, Kocaeli 41001, Türkiye; § Adıyaman Univesity, School of Medicine, Department of Biophysics, Adıyaman 02040, Türkiye; ∥ 52980 Kocaeli University, Department of Chemistry and Chemical Processing Technologies, Kocaeli 41140, Türkiye; ⊥ Bahcesehir Univesity, School of Medicine, Department of Biophysics, Istanbul 34734, Türkiye

## Abstract

Herein, PLA/TH/PEG electrospun nanofibers were fabricated by incorporating tetrabutylammonium hexafluorophosphate (TH) salt at different ratios (3, 5, 7, and 9 wt %) into the poly­(lactic acid) (PLA)/poly­(ethylene glycol) (PEG) matrix. Thus, the potential use of PLA/PEG/TH nanofibers with enhanced properties as dermal wound dressings was evaluated. Cytotoxicity and wound healing assays using the fibroblast cell line (L929) were performed. An increase in TH content tended to reduce the wound healing rate, with decreases ranging from approximately 1.7% to 45% compared with PLA/PEG nanofibers. However, it was concluded that PLA/TH/PEG nanofibers did not cause toxic effects and even supported cell proliferation in the range of 113–123%. The produced PLA/TH/PEG nanofibers were identified as being suitable for dermal wound dressings. When TH was added, the beaded surface structure observed in the PLA/PEG nanofibers improved and the nanofiber diameters decreased. The PLA/TH9/PEG nanofibrous material exhibited the most homogeneous and smooth nanofiber morphology. Also, it had the lowest fiber diameter and the highest porosity (90%) according to the porosity test. Consequently, it is suggested that TH is suitable for use as an additive in biomedical applications owing to its nontoxic properties.

## Graphical abstract

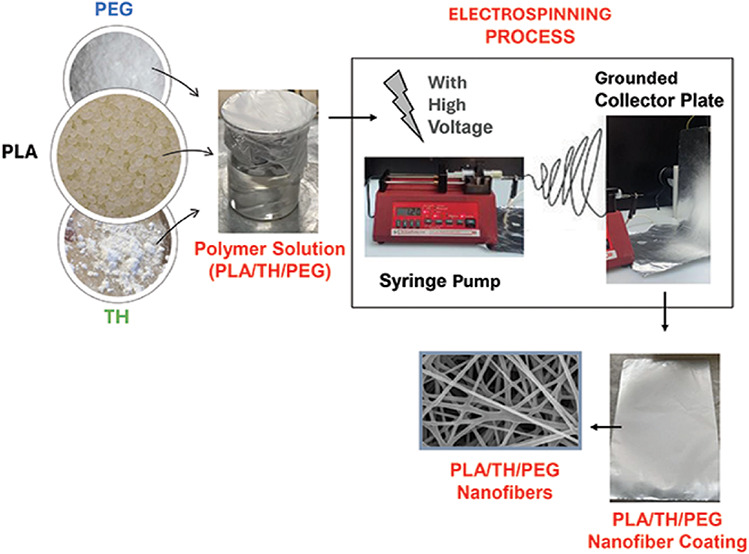

## Introduction

The skin, the largest organ in the human body, protects other organs from external damage. However, it is always open to environmental threats. Wound dressings protect the injured skin surface from external effects and prevent further damage. An ideal wound dressing should not only copy the extracellular matrix’s (ECM) structure but also replicate many functions that affect cell behavior, such as adhesion, growth, and proliferation.^1,2^ A useful wound dressing is expected to have features such as high air permeability, the ability to support cell growth and rapid hemostasis, and the ability to keep the wound moist.^3^ In particular, the moisture balance of the wound is critical for wound healing, as moisture facilitates the migration of new skin cells from the edges of the wound, resulting in faster wound closure.^4^ Many skin substitutes, such as xenografts, allografts, and autografts, are used for wound healing. However, these approaches have disadvantages such as high cost, limited availability of skin grafts for advanced burn cases, inevitable complications on the wound surface, and low immune response problems.^5^ Nowadays, to cope with serious skin damage, skin replacement studies produced with tissue technology are being carried out.

In recent years, various wound dressing forms, such as nanofibers, sponges, and hydrogels, have been designed to accelerate wound healing. Since nanofiber membranes resemble the natural extracellular matrix (ECM), they are capable of supporting cell proliferation and migration.^3^ Micro/nano fibers provide effective contact with the wound surface, thanks to their high porosity and high surface-to-volume ratio. This feature increases the bioavailability of wound healing compounds.^4,6^ Electrospinning is an effective technique to produce nanofibrous wound dressing material forms. Using this technique, tissue scaffolds with micro/nanoscale fibers can be produced from synthetic and natural polymers.^5,7,8^

In biomedical fields, biopolymers such as poly­(lactic acid) (PLA), poly­(butylene succinate) (PBS), poly­(hydroxy butyrate) (PHB), poly­(vinyl alcohol) (PVA), and poly­(caprolactone) (PCL) are frequently used.^9–11^ Due to its brittle behavior, it has been stated in the literature that PLA is often used with plasticizers (e.g., glycerol, citrate esters) due to its structural properties.^12^ Apart from these, PLA is mixed with nontoxic, flexible, and hydrophilic polymers such as poly­(ethylene glycol) (PEG) and poly­(ethylene oxide) (PEO).^8,13^ Thus, the plasticizing effect of these polymers is utilized. It has been stated in the literature that PEG is compatible with PLA and that PEG has been used as a suitable plasticizer for PLA in many studies.^8,14–16^

Electrospun PLA fiber constructs are expected to have a positive effect on L929 cell adhesion, proliferation, and migration, making them suitable for tissue engineering and wound healing applications. A study by Yang et al. showed that electrospun PLA fibers promoted the adhesion and proliferation of fibroblasts and that the fibers acted as scaffolds mimicking the native extracellular matrix (ECM).^17^ Additionally, the porous structure facilitates nutrient exchange and promotes cell growth. Ramakrishna et al. showed that fibroblast cells seeded on electrospun PLA fibers exhibited higher proliferation rates compared to those on flat PLA films. The increased surface area of the nanofibers provided more areas for cells to adhere, increasing their proliferation.^18^ The porosity and surface properties of the fibers provide a biomimetic environment that supports cell growth, while the biodegradable structure of PLA offers additional benefits such as controlled drug release. These properties have been demonstrated in numerous studies on different cell lines, including fibroblasts, demonstrating the versatility of electrospun PLA in biomedical applications. Wound healing and increased cell viability can be achieved by improving the electrospun nanofiber structure. Salt structures can be added to the electrospun solutions to improve the surface properties of the nanofibers. In the study conducted by Topuz et al., the effect of Tetraethylammonium bromide (TEAB) salt addition on nanofibers of microporous polyimides via electrospinning was investigated and it was proven that the presence of salt led to the formation of thinner electrospun fibers.^19^ In our previous study, it was shown that the beaded structure seen in pure PLA/PEG nanofibrous material disappeared with the addition of quaternary salt to the PLA/PEG matrix.^20^ In another study, Hemmat et al. proved that porosity increased with the addition of salt in nanofiber membranes produced with different salt additives.^21^

The complete category of cationic surfactants features a distinct structure characterized by a positively charged hydrophilic head and a hydrophobic tail, typically composed of a long alkyl chain within a single molecule. Based on variations in the structure of the hydrophilic head, cationic surfactants can be classified into four groups: amines, quaternary ammonium compounds, sulfonium salts, and phosphonium salts. Tetrabutylammonium hexafluorosulfate (TBAHP) salt was used in the study by Li et al. TBAHP, which is from the quaternary ammonium family, is a fluoride salt with strong hydrophobicity. At the same time, TBAHP is an organic branched salt that can be easily dissolved in organic solvents and can significantly increase the conductivity of the electrospinning solution.^22^ In addition to these properties, it has biological effects that are high in antibacterial activity according to some studies in the literature and low in some studies.^23,24^ Li et al. investigated the antibacterial activity by a qualitative method and found no activity. A PVDF/TBAHP/PS MNM with superhydrophobicity, improved thermal efficiency, and high porosity was successfully designed and developed via one-step electrospinning.^22^ It also has a structure that can show toxic effects as the TH salt concentration increases.^23,25^ However, its potential effects on biological systems, such as the L929 cell line (fibroblasts), have not been extensively investigated.

Recent advances in electrospinning technology have focused on improving fiber uniformity, structural stability, and functional performance through solution parameter optimization and the incorporation of ionic or bioactive additives. Modulation of solution conductivity, viscosity, and intermolecular interactions has emerged as an effective strategy to tailor nanofiber morphology and enhance structure–property relationships.^26,27^ In particular, salt-assisted electrospinning has been widely explored to control jet stability and fiber diameter distribution. However, the functional performance of electrospun wound dressings is often strongly dependent on the nature and behavior of the incorporated active components. While many studies report antimicrobial or bioactive enhancements, the final biological outcome is governed by the molecular structure, dispersion state, and surface availability of the loaded additive.^28,29^ In this context, investigating the role of tetrabutylammonium hexafluorophosphate (TH) is particularly relevant, as its ionic characteristics may influence not only fiber morphology and thermal stability but also cell–material interactions.^30^ Therefore, this study aims to elucidate how TH incorporation affects the structural and biological performance of PLA/PEG nanofibrous wound dressing systems.

In this study, PLA and PEG were mixed in appropriate proportions and processed by the electrospinning method according to the information obtained from our previous studies.^7,8,20^ TH salt was added to the PLA/PEG mixture at different additive ratios in order to improve the physical, chemical, and biological properties of the nanofibrous surface and to be used as a dermal wound dressing. Characterization studies of PLA/TH/PEG nanofibers were carried out. In particular, apart from the qualitative studies available in the literature, antibacterial activity examination with a quantitative method, evaluation of L929 fibroblast cell viability with a direct cytotoxicity test method, and wound healing investigation studies with the Assay Method in the L929 fibroblast cell line under in vitro conditions were carried out. This study covers qualified results for the usability of TH salt in biomedical applications.

## Material and Method

### Material

In this study, polylactic acid (PLA) (Lx175) (Mw: 128.49 kDa) was purchased from the Luminy brand, and poly­(ethylene glycol) (PEG) (Mw: 2000 g/mol) was purchased from the Fluka brand. Tetrabutylammonium hexafluorophosphate as a quaternary ammonium salt was procured from Merck. In the preparation of the polymer solution, chloroform (CF, CHCl_3_, Baker, 99%), dichloromethane (DCM, CH_2_Cl_2_, Carlo-Erba, 99.9%), and N,N-dimethylformamide (DMF, (CH_3_)_2_NC­(O)­H, ISOLAB, Extra Pure) solvents were used.

### Preparation of Solutions and Electrospinning Process

The PLA polymer was primarily dried under a vacuum at 55 °C for 12 h to remove humidity. According to the data obtained from our previous study, a 7 wt % PLA concentration was prepared. 20 wt % PEG was added by weight according to the amount of solid PLA. Thus, a PLA/PEG mixture was obtained. The TH additive to be added was also calculated and added at 3%, 5%, 7%, and 9 wt % according to the amount of solid PLA. The solutions in this study were obtained according to the procedure in our previous study. The polymer solutions prepared for the electrospinning process were applied without waiting. According to our previous study, the electrospinning process was carried out under the same electrospinning and environmental conditions. Briefly, the polymer solutions were dissolved in 10 mL of a DCM/DMF/CF solvent mixture (7/2.5/0.5, v/v/v) and stirred for 3 h at room temperature in an airtight beaker to ensure complete homogenization. Electrospinning was performed inside a closed chamber without airflow using a 22G × 1 in. stainless steel needle. The applied voltage was 17 kV; the solution flow rate was maintained at 1.5 mL/h, and the needle-to-collector distance was set at 15 cm. The ambient temperature was controlled at 25.5 °C with a relative humidity of approximately 40%. Each solution was electrospun for 2 h.^7,8,20^ The average nanofiber surface thickness was measured as 0.20 mm using a caliper.

### Characterization

The IR spectra in the range of 650–4000 cm^–1^ of TH-loaded PLA/PEG nanofibrous materials were examined with a PerkinElmer Spectrum 100 FTIR device with an ATR unit spectrometer.

Differential scanning calorimetry (DSC) analysis with the Mettler Toledo DSC 1 device was conducted at a heating rate of 10 °C/min within the temperature range of 25 to 300 °C in a single step.

The degree of crystallinity (*X*_*c*_) of fibers was calculated using the (Δ*H*_*m*_^0^) value of PLA from the results of the DSC analysis with the following equation. The (Δ*H*_*m*_^0^) value of PLA was used as 93.7 J/g obtained from our previous studies,^7^ where Δ*H*_*m*_ is the heat of melting of each sample, Δ*H*_*c*_ is the crystallization enthalpy, ω_*f*_ is the weight fraction, and Δ*H*_*m*_^0^ is the heat of melting of the matrix. *X*_c_ was calculated according to “eq 1”:

1 $$X_{c\;}\left(\%\mathrm{crystallinity}\right)=\frac{\Delta H_{m}-\Delta H_{c}}{\left(\omega_{f}\right)\times \Delta {H_{m}}^{0}}$$

Thermal gravimetric analysis (TGA) in the temperature range between 25 and 600 °C and a heating rate of 10 °C/min was performed with a Mettler Toledo TGA 1 device. High-purity nitrogen gas was sent to the system at a flow rate of 30 mL/min during DSC and TGA analyses.

Liquid absorption capacity (%) of nanofibrous materials was carried out with the EDANA 10.3.99 standard according to our previous studies under environmental conditions.^7^ Liquid absorption capacity (LAC) of nanofibrous materials according to the initial dry weight and subsequent wet weight ratio (%) was calculated using “eq 2”:

2 $$\mathrm{LAC}\left(\%\right)=\frac{N_{s}-N_{k}}{N_{k}}\times 100$$

where *N_s_* denotes the weight of the wet nanofibrous materials and *N_k_* represents the initial dry weight of the nanofibrous materials. Each sample was tested five times, and the average of the recorded values was analyzed. The calculated standard deviation values are presented as error bars on the LAC column graph in the Result and Discussion section.

The porosity of the nanofibrous material was determined using absolute ethanol according to the Archimedean principle^31,32^ (liquid displacement method), as outlined in “eq 3”:

3 $$\mathrm{Porosity}\;\left(\%\right)=\left[\frac{\left(W_{2}-W_{3}-W_{s}\right)}{\left(W_{1}-W_{3}\right)}\right]\times 100$$

*W*_1_ represents the weight of the beaker containing only pure ethanol; *W*_2_ denotes the weight of the beaker filled with pure ethanol and nanofibers; *W*_3_ refers to the weight of the beaker and pure ethanol after the ethanol-wetted nanofibers have been removed from *W*_2_; and finally, *W*_*s*_ indicates the dry weight of the nanofibers. The average of three samples was measured, and the standard deviation values are shown as the error bars on the porosity column graph in the Result and Discussion section. The porosity values were not derived from a 2D SEM image analysis. As part of the investigation, images of PLA nanofibrous materials with TH salt additives were captured by using a JEOL brand JSM-6060 model scanning electron microscope. The average fiber diameter of the PLA/PEG and TH-loaded PLA/PEG nanofibrous materials was analyzed by using ImageJ software from the SEM images. The diameter measurements of the nanofibers were calculated by averaging 30 strands, and error bars represent the standard deviation.

### Cytotoxicity Analysis

The cytotoxicity of the nanofibrous materials was assessed through a direct contact test using the WST-1 assay on a mouse fibroblast cell line (L929, ATCC, and CCL-1). Cell viability (%) was determined according to “eq 4”:^7,8^

4 $$\mathrm{Cell vailabilty \%}=\frac{A_{b\;\mathrm{sample}}-A_{b\;blank}}{A_{b\;control}-A_{b\;blank}}\times 100$$

*A*_*b sample*_ represents the absorbance of the nanofibrous material; *A*_*b blank*_ denotes the absorbance of the blank sample; and *A*_*b control*_ indicates the absorbance of the negative control. Three nanofibers were measured; the average was calculated, and the standard deviation values were shown as the error bars on the cell viability column graph in the Result and Discussion section.

### Wound Healing Analysis

This study was carried out in the cell laboratory of the Department of Biophysics, Faculty of Medicine, Bahçeşehir University. One million L929 cells were seeded into a 25 cm^2^ vial and incubated for 24 h. Following incubation, the cell density in the flask was verified, and upon confirmation of full confluence, a wound model was induced with a cell scraper. Fresh medium was then added to replace the old medium. Nanofiber samples (10 × 10 mm) were prepared and then sterilized before the experiment. They were finally kept in the flask to see whether they helped in wound healing. The healing process of the wound-created model was monitored every 5 min for 2 days using the Celloger Nano device. The experiment was conducted under standard conditions where cells were incubated at 37 °C, 5% CO_2_, and 95% relative humidity, and reported wound closure rates were calculated from continuous monitoring data. The wound healing rate testing was fulfilled with three nanofibers. In the Result and Discussion section, wound healing images were presented by selecting the best captured images. For the numerical values used in the bar graph, the average was calculated, and the standard deviation of the values was given with error bars.

### Antibacterial Test

Agar plate colony counting, a quantitative method, was employed to evaluate the antibacterial properties of TH-loaded PLA/PEG nanofibrous materials. This test was conducted following the AATCC-100 standard for 24 h, as detailed in our previous study.^7,8^ The standard strains of *Escherichia coli* (ATCC 8739) and *Staphylococcus aureus* (ATCC 6538P) were used for antibacterial analysis. Both bacteria were cultured in a Nutrient Agar medium (BioLife, Italy). Before initiating the studies, the surfaces of the samples were sterilized with UVC for 30 min in the biosafety cabinet.

Antibacterial activity of nanofibrous materials is calculated according to “eq 5”:

5 AntibacterialActivity(%)=(A−B)/A×100

where *A* represents the average colony count of the untreated nanofibrous material control sample; and *B* indicates the average number of colonies per hour, excluding the 0-h count with the fewest colonies. The antibacterial test was repeated with three samples. Appropriate images were selected to match the test results and added to the Result and Discussion section.

## Result and Discussion

### FT-IR Analysis of Nanofibrous Materials

FTIR spectra of the TH salt, PLA/PEG, and PLA/TH/PEG nanofibrous materials are given in Figure 1. When the spectrum of TH salt was examined, the peaks at 2969 cm^–1^ and 2877 cm^–1^ represented the asymmetric and symmetric stretching of sp^3^–CH_2_ and −CH_3_, while the peak at 1471 cm^–1^ was associated with the in-plane bending vibrations of methyl and methylene groups.^33^ The peak observed at 1167 cm^–1^ was attributed to the (C–F) bond.^34^ The peak at 827 cm^–1^ was attributed to the (C=CH) group. When the FT-IR spectrum of PLA/PEG nanofibers was examined, the prominent peak at 1757 cm^–1^ attributed to PLA indicated the presence of the (C=O) group, and the peak at 1180 cm^–1^ (C–O–C) indicated the presence of strong ester groups. In addition, it was observed that the peak at 1453 cm^–1^ corresponded to the CH_3_ bending vibration associated with the molecular structure of PEG. The distinct peak at 1084 cm^–1^, which is characteristic of PEG, confirmed the presence of the (C–O) group. In addition, the peaks at 962 cm^–1^ and 840 cm^–1^ were associated with the (−CH=CH−) group. All of these peaks, which are characteristic of the PLA/PEG matrix, were observed in the FTIR spectra of PLA/TH/PEG nanofibers. However, a decrease in the intensities of these peaks was observed due to the presence of the TH salt. The characteristic peaks of the TH salt were not observed in PLA/TH/PEG nanofibrous materials. This was associated with the TH salt acting as a catalyst in the solution for the electrospinning process. For this reason, it is normal that the characteristic peaks of the TH salt were not observed in the FT-IR spectra of PLA/TH/PEG nanofibers. It has been confirmed in the literature that any salt added to the solution enables the improvement of nanofiber properties by changing the solution parameters (conductivity, viscosity, etc.) and electrospinning production conditions (applied voltage, feed amount, etc.).^35–37^

**Figure 1:**
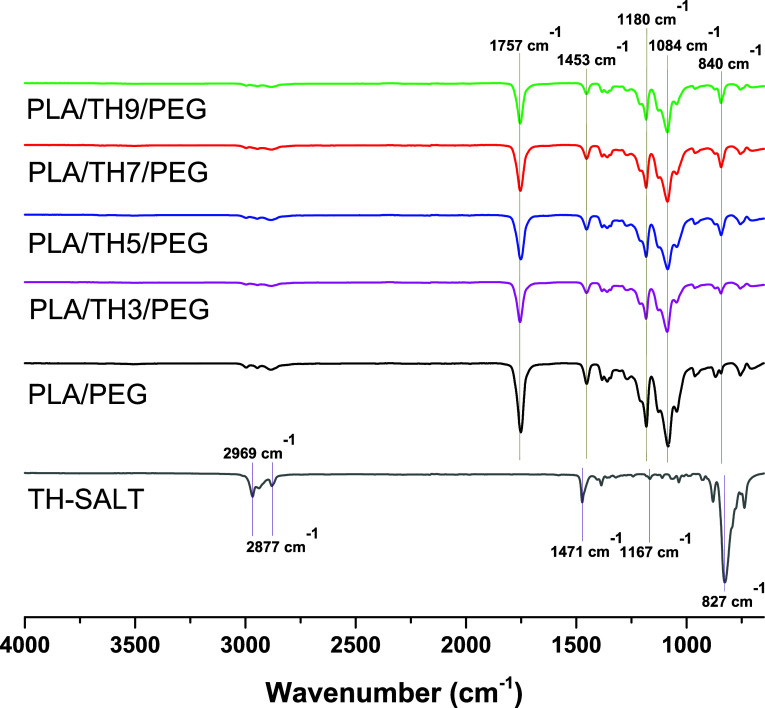
FT-IR spectra of hollow nanofibrous materials.

Although the characteristic peaks of the TH salt were not distinctly observed in the FTIR spectra of PLA/TH/PEG nanofibrous materials, this does not necessarily indicate the absence of TH within the matrix. The relatively low concentration of TH (3–9 wt %) and its ionic nature may result in peak overlapping or masking by the dominant PLA and PEG absorption bands. In addition, ionic interactions between TH and the polymer chains may reduce the intensity of the characteristic TH vibrations. The successful incorporation of TH into the nanofibrous structure is indirectly supported by the increased residual content observed in TGA analysis, the variation in crystallinity values, and the significant morphological changes in the fiber diameter and bead structure. These combined findings provide strong evidence of the presence and effective dispersion of TH within the PLA/PEG matrix.

### DSC-TGA Analysis of Nanofibrous Materials

The data on the thermal properties of TH salt-added PLA/PEG nanofibrous materials and the values of their degradation temperatures are given in Table 1. In the literature, the T_g_ temperature of pure PLA nanofibers is reported as approximately 64 °C, the T_c_ temperature as approximately 87 °C, and the T_m_ temperature as approximately 155 °C.^38^ Similarly, in the literature, the T_c_ temperature of pure PEG nanofiber is stated as 25 °C and the T_m_ temperature as 50 °C.^39^ According to the DSC test results, the melting temperatures of the TH salt used in the study were measured as 36.91 °C, 93.07 °C, and 250.77 °C, respectively. The T_m1_ and T_m2_ temperatures of the PLA/PEG nanofiber were determined as 52 and 156 °C, respectively. The T_m1_ temperature was attributed to the melting temperature of PEG, while the T_m2_ temperature was associated with the melting temperature of PLA. The energy released due to the presence of T_m1_ has absorbed the T_g_ temperature energy of pure PLA. For this reason, while the T_g_ temperature of PLA/PEG nanofibrous material was not observed, the crystallization temperature gave a single T_c_ value of 66 °C (6.14 J/g). Crystallization temperatures were not observed in TH salt-doped nanofibrous materials. It can be said that the T_m_ temperature energies of TH salt have absorbed the T_c_ temperature energy.^20^ While the T_m1_ temperature of PLA/TH/PEG nanofibrous materials was observed in the range of 53–54 °C on average, the T_m2_ temperatures were observed in the range of 154–156 °C. It has been reported that the TH salt doping did not affect the melting temperatures of the PLA/PEG matrix. It was observed that the % crystallinity values of PLA/TH/PEG nanofibrous materials increased up to 5 wt % TH salt-doped nanofibrous material compared to pure PLA/PEG nanofibers and then decreased. This decrease is attributed to the irregular arrangement of salt ions on the polymer chain, which increases branching and changes the structural orientation. In addition, the presence of salt ions can increase the distance between polymer chains, cause voids, and disrupt the crystalline structure by reducing the interaction between polymer chains. The T_deg5_, T_deg10_, and T_deg50_ degradation temperatures of pure PLA/PEG nanofiber were reported as 300.13 °C, 308.94 °C, and 336.94 °C, respectively. An increase in these temperatures was observed in the TH salt-doped PLA/TH/PEG nanofibrous materials. Especially, the T_deg5_ temperatures of PLA/TH3/PEG and PLA/TH5/PEG nanofibers were found to be 324.87 and 306 °C, respectively. As a result, it was reported that TH salt doping increased the thermal resistance of nanofibrous materials. According to the residue amounts, a linear increase was observed with the TH salt doping. All nanofibers exhibited approximately an 8% (±1) residue amount.

**Table 1:** Thermal Features and Degradation Values of Nanofibrous Materials

Samples	T_m1_ (^o^C)	T_m2_ (^o^C)	ΔH (J/g)	X_c_ (%)	T_deg5_	T_deg10_	T_deg50_	Residue (%)
**PLA/PEG**	52.57	156.38	28.48	29.8	300.13	308.94	336.94	7.06
**PLA/TH3/PEG**	54.10	156.93	23.41	31.2	324.87	334.94	354.03	7.22
PLA/TH5/PEG	53.77	154.77	25.56	34.1	306.00	334.94	360.59	7.91
**PLA/TH7/PEG**	54.30	154.82	18.56	24.5	277.48	327.39	354.03	7.99
**PLA/TH9/PEG**	53.66	156.50	18.93	25.2	269.93	314.39	355.64	8.72

TGA results revealed a concentration-dependent thermal behavior. At lower TH contents (3 and 5 wt %), the onset degradation temperatures (T_deg5_ and T_deg10_) increased compared to neat PLA/PEG, which may be attributed to enhanced ionic interactions and increased crystallinity that restrict polymer chain mobility. However, at higher TH loadings (7 and 9 wt %), a reduction in T_deg5_ was observed. This behavior may be associated with excessive ionic aggregation and microvoid formation, leading to localized structural defects. Despite these variations in early-stage degradation, T_deg50_ values remained higher than that of neat PLA/PEG, indicating that the bulk thermal stability of the nanofibrous matrix was not adversely affected. The gradual increase in residual content further supports the presence of thermally stable inorganic components within the matrix.

### SEM Micrographs of Nanofibrous Materials

Average fiber diameter values and SEM micrographs of PLA/TH/PEG nanofibrous materials are given in Figures 2 and 3, respectively. The diameter measurements of the nanofiber surfaces were added by averaging 30 strands, and error bars represent the standard deviation. When the SEM surface images are viewed, the beaded surface structure of the pure PLA/PEG nanofibrous material stands out. It has been reported that by adding TH salt to the structure, the beads disappear, and the surface images of all nanofibers improve. With a 3 wt % TH additive, the solution viscosity increased, but the applied constant voltage was insufficient for homogeneous fiber formation. For this reason, occasional adhesions were observed between PLA/TH3/PEG nanofibrous materials. Due to high viscosity and insufficient voltage, the fibers tended to stick. It was observed that 5 and 7 wt % TH-doped nanofibrous materials exhibited similar surface images, and their diameters became thicker. The increase in viscosity of the solution prepared with the addition of 5 wt % TH caused the formation of the thickest fibers, with a value of 468 nm. While TH salt addition increased the solution viscosity, it also increased the solution conductivity.^40,41^ It has been confirmed in the literature that the addition of salt to the polymer matrix causes an increase in solution conductivity.^8,35^ However, the solution conductivity in the face of a constant voltage was insufficient for regular fiber formation, resulting in PLA/TH5/PEG nanofibers. It has been stated that this thickens the fibers and causes adhesions. It has been observed that the fibers tend to become thinner as a result of the increase in solution viscosity and conductivity with the addition of 7 wt % TH salt. It has been noticed that the voltage which is applied at a constant value is sufficient for the solution, whose conductivity increases and surface tension decreases, to form homogeneous fibers. However, since the electrospinning production conditions are not fully sufficient to create fibers with optimum properties, it has been observed that the fiber diameter and thickness of the PLA/TH7/PEG nanofibrous material are higher than those of other nanomaterials but lower than those of the PLA/TH5/PEG nanofibrous material. The most optimum electrospinning production conditions and solution parameters for obtaining homogeneously distributed and thin fibers with a smooth surface appearance and no interfiber adhesion were noticed in the 9 wt % TH-doped nanofibrous material. It has been reported that among TH salt-doped nanofibers, PLA/TH9/PEG nanofibrous material has the thinnest diameter fibers with a value of 306 nm.

**Figure 2:**
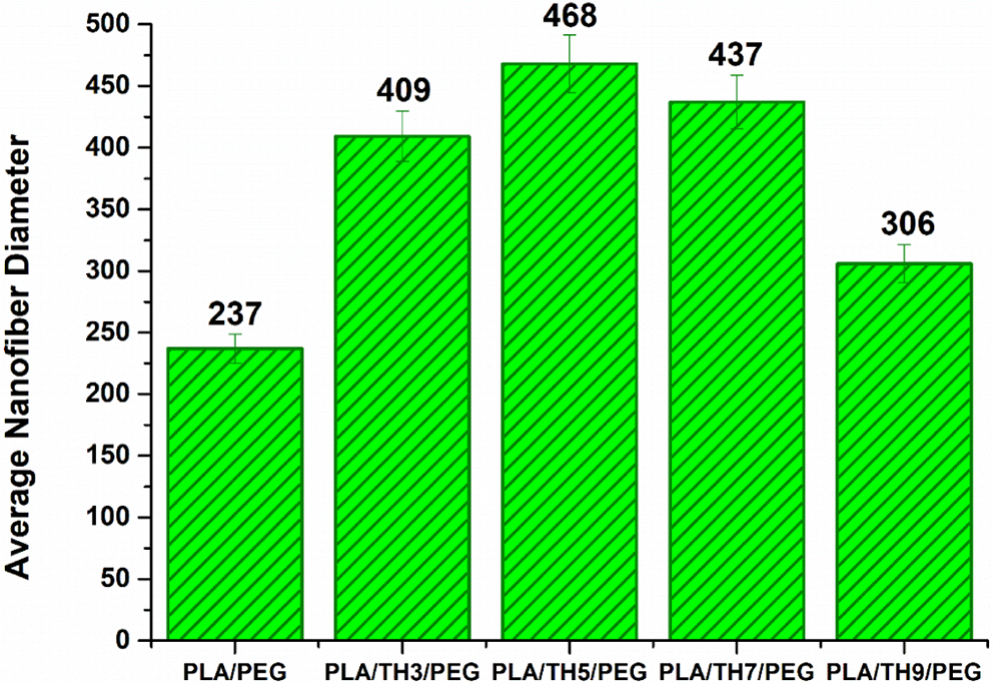
Average nanofiber diameters of nanofibrous materials (nm).

**Figure 3:**
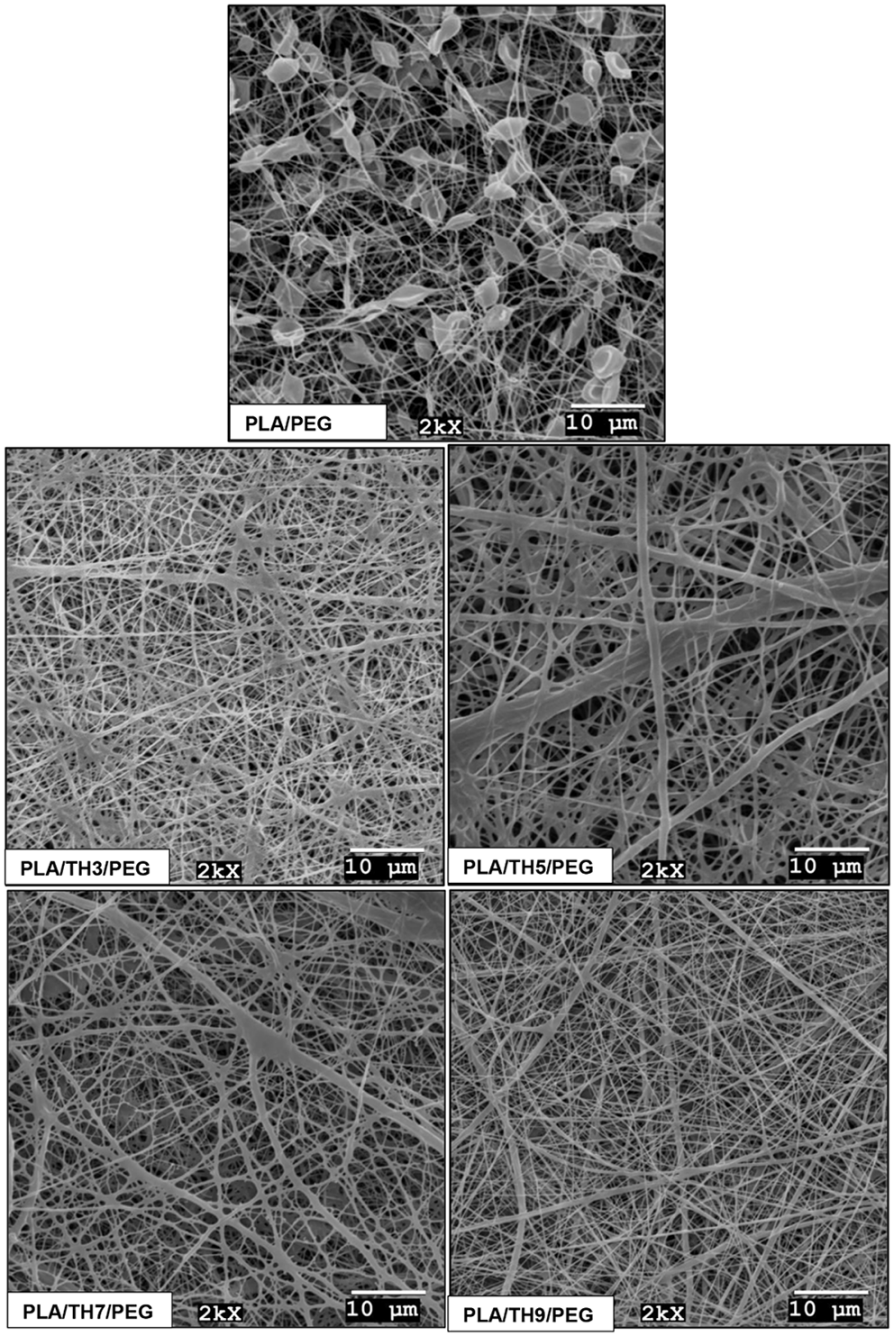
SEM micrographs of nanofibrous materials (mag: 2000×, scale: 10 μm).

### Porosity Analysis of Nanofibrous Materials

The porosity (%) values of the produced PLA/PEG and TH salt-added PLA/PEG nanofibrous materials are given in Figure 4. The PLA/PEG nanofibrous material was shown to have the highest porosity, with a value of 95%. This value can be associated with the fact that PLA/PEG nanofibers have the thinnest fiber diameter values, because it is known that porosity increases with decreasing fiber diameter.^42^ However, in this study, it was noticed that there was no linear relationship between porosity and fiber diameter due to the characteristic effects of the TH salt added to the PLA/PEG matrix. A decrease in porosity values was observed with the addition of the TH salt to the PLA/PEG structure. It was observed that the PLA/TH9/PEG nanofibrous material had the highest porosity compared with other TH salt-added nanofibers. In the literature, it is considered sufficient for the materials to be used as wound dressings to have 60–90% porosity.^43^ Accordingly, it has been reported that there is no problem in using all of the nanofibrous materials obtained as wound dressings. When PLA/TH/PEG nanofibrous materials are used as wound dressings, it is thought that while protecting the wound from external factors, contact with air will not also be interrupted, owing to their pores. It is predicted that this situation will contribute to the rapid healing of the wound.

**Figure 4:**
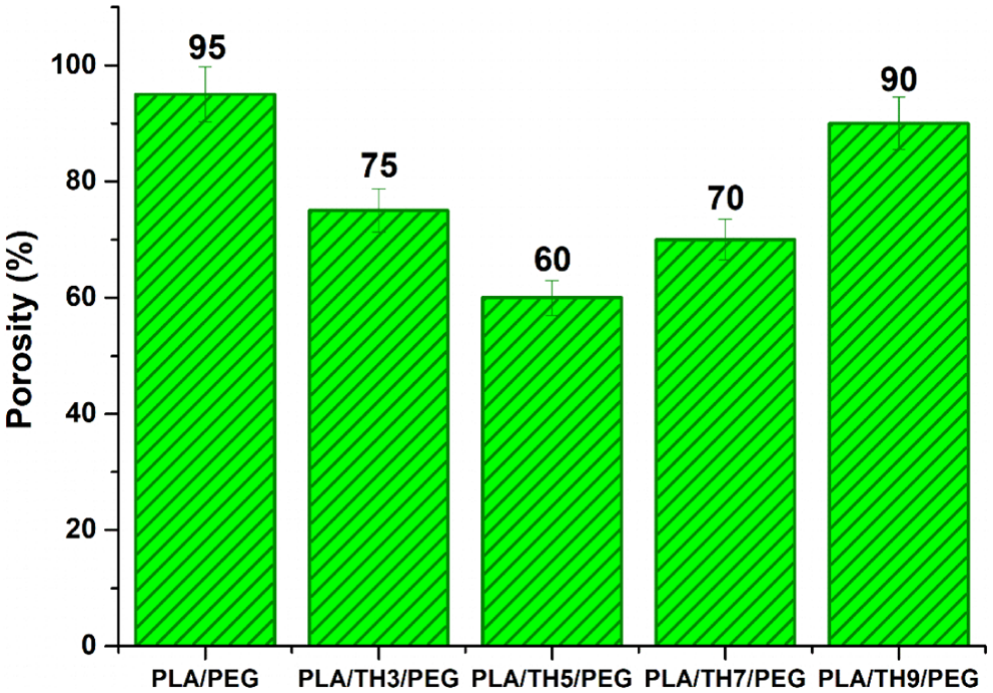
Porosity (%) results for all nanofibrous materials.

### Analysis of Liquid Absorption Capacity of Nanofibrous Materials

Liquid absorption capacities (LAC, %) of PLA/PEG and PLA/TH/PEG nanofibrous materials are given in Figure 5. According to the liquid absorption test results, the PLA/PEG nanofibrous material was reported to have a capacity of 292.5%. While hydrophilic-structured PEG absorbs water molecules, hydrophobic-structured PLA repels them. Therefore, the LAC behavior of the PLA/PEG nanofibrous material was associated with the desire for water absorption of PEG. With the addition of TH salt, PLA/TH3/PEG and PLA/TH5/PEG nanofibrous materials exhibited increased liquid absorption capacities, with values of 442.5% and 555.6%, respectively. This situation can be associated with the tendency of fluorine groups in the TH salt structure to interact with water molecules when they encounter them.^44^ However, after the addition of 5 wt % TH salt, the liquid absorption capacity of PLA/TH7/PEG and PLA/TH9/PEG nanofibers decreased to approximately 342% (±1) regardless of the increasing salt amount, and the LAC value remained constant. The 5 wt % addition of TH salt was evaluated as the saturation point in the PLA/PEG matrix. The distribution and local aggregation of salt ions in the polymer matrix reduced the LAC behavior. The liquid absorption behavior of nanofibrous materials was associated with the tendency of salt ions to absorb water rather than being specifically related to the percentage of porosity. As a result, it was reported that the hydrophilic TH salt improved the liquid absorption capacity of the pure PLA/PEG nanofiber. It is a desired feature for the environment to remain moist for wound healing.^45^ Therefore, it is expected that a wound dressing will absorb 100–900% liquid.^46,47^ In this study, it was reported that all nanofibrous materials produced can be used as wound dressings.

**Figure 5:**
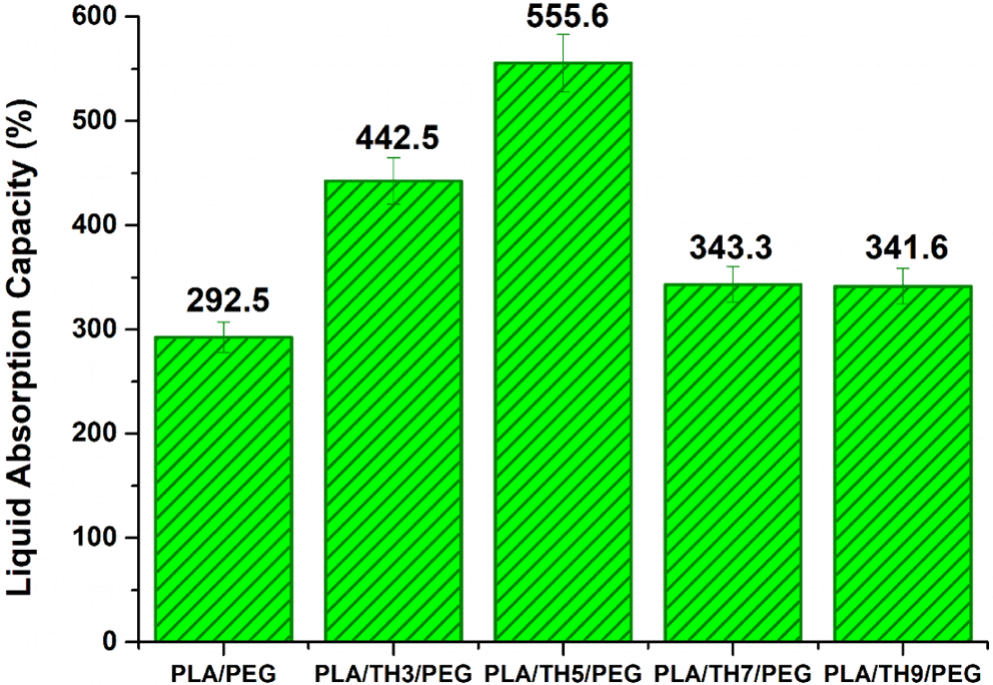
Liquid absorption capacity (%) of raw nanofibrous materials.

### Cytotoxicity Analysis of Nanofibrous Materials

The cell viability (%) of all produced nanofibrous materials was evaluated with a cytotoxicity test. Cell viability values of pure PLA/PEG and PLA/TH/PEG nanofibrous materials at the end of 24 h are shown in Figure 6. A cytotoxicity test was also carried out for 24 h, correlating with the duration of the antibacterial test. The PLA/PEG nanofibrous material exhibited fibroblast cell viability with a value of 93.39%. A sharp increase in cell viability values is observed with the addition of TH salt to the structure. The cell viability of nanofibrous materials with TH salt added increased by approximately 21–31% compared to pure PLA/PEG nanofiber. Cell viability in PLA/TH3/PEG and PLA/TH5/PEG nanofibrous materials was observed to be close to each other with a value of about 114%. It is known that cell adhesion increases as the fiber diameter of nanofibrous materials becomes thinner.^48^ According to the cytotoxicity test results in this study, it was observed that there was no linear relationship between cell viability and fiber diameter. It was emphasized that the TH salt directly acts as a nutrient for fibroblast cells. It was reported that the TH salt was not toxic. Thus, as the amount of TH salt increased, the proliferation of the fibroblast cells also increased. It is thought that this will accelerate wound healing by providing cell propagation. When evaluated in terms of patient comfort, it is predicted that the rapid closure of wounds will allow the patient to adapt to their daily activities in a short time. Based on the ISO 10993-5 standard, a material with a cell viability value of 70% or greater may be utilized as a wound dressing.^49^ It has been reported that all PLA/TH/PEG nanofibrous materials can be safely used as wound dressings according to this standard. It has been stated that the PLA/TH9/PEG nanofibrous material has the highest cell viability, with a value of 122.67% at the end of 24 h due to both the thinnest fiber diameters and the maximum TH salt contribution.

**Figure 6:**
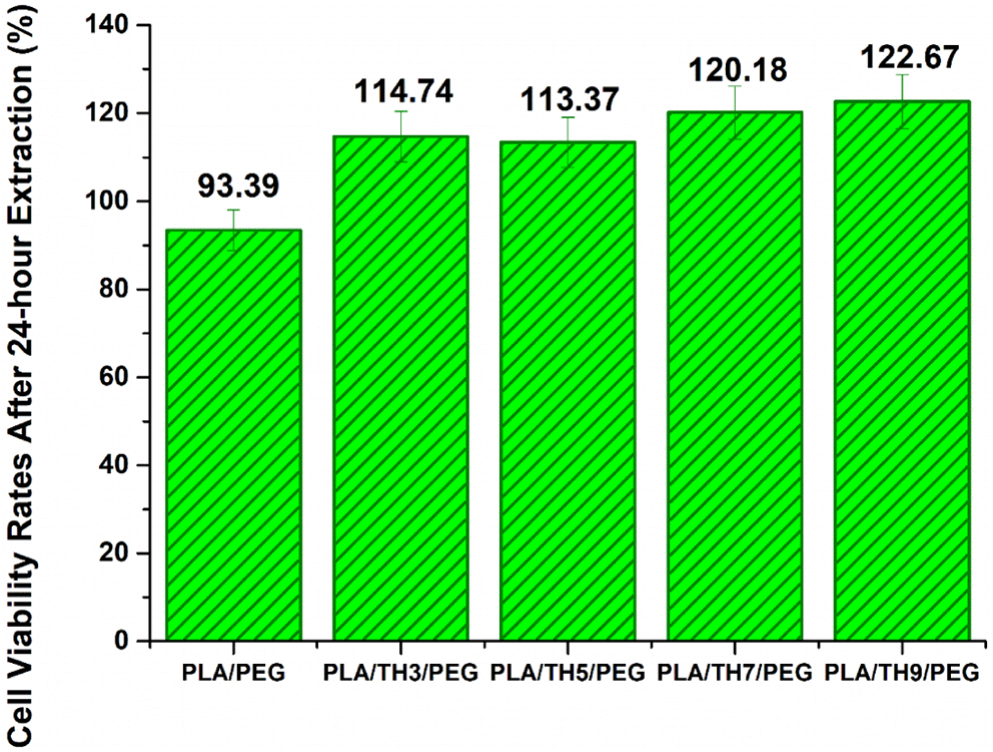
Cytotoxicity results (%) of nanofibrous materials.

### Wound Healing Analysis of Nanofibrous Materials

As stated in the literature, PLA is a suitable biomaterial for the proliferation of fibroblast cells.^17,50^ In addition, since nanofibers are similar to ECM in nature, they are expected to accelerate wound healing. In this study, it was predicted that nanofibers obtained from PLA and its derivatives would exhibit superior properties when used as skin wound dressings. The time-dependent wound healing column graph and images of PLA/PEG and PLA/TH/PEG nanofibrous materials are given in Figure 7 and Table 2, respectively. Pure PLA/PEG and PLA/TH3/PEG nanofibrous materials exhibited a closing speed of 7.11 and 6.99 μm/h, respectively. However, a sharp decrease in the wound healing rate of PLA/TH/PEG nanofibers was observed with an increasing TH salt amount in the structure. According to the cell viability test results, the TH salt acts as a nutrient for fibroblast cells and supports the proliferation of cells. However, while fibroblast cells proliferate rapidly in the presence of salt, TH salt particles settled along the nanofibrous surface limit the mobility of fibroblasts and slow their migration to the wound area. It was noticed that the fibroblast wound healing rate decreased when TH salt was used at more than 3 wt %. Since there were no salt agglomerations that could prevent fibroblast migration in pure PLA/PEG nanofiber, it provided maximum rapid wound closure performance.

**Table 2:**
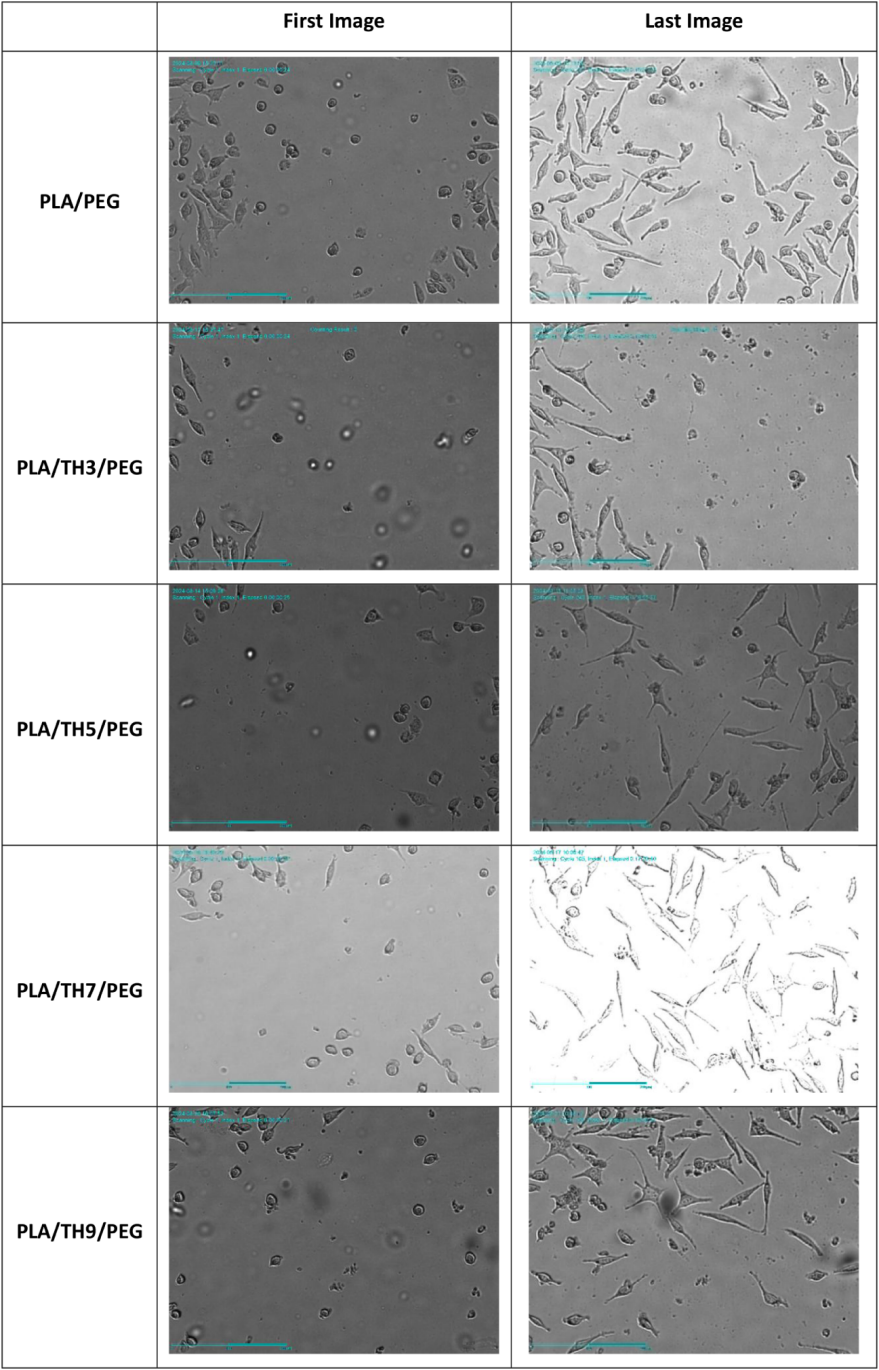
Time-Dependent Wound Closure Images of Nanofibrous Materials

**Figure 7:**
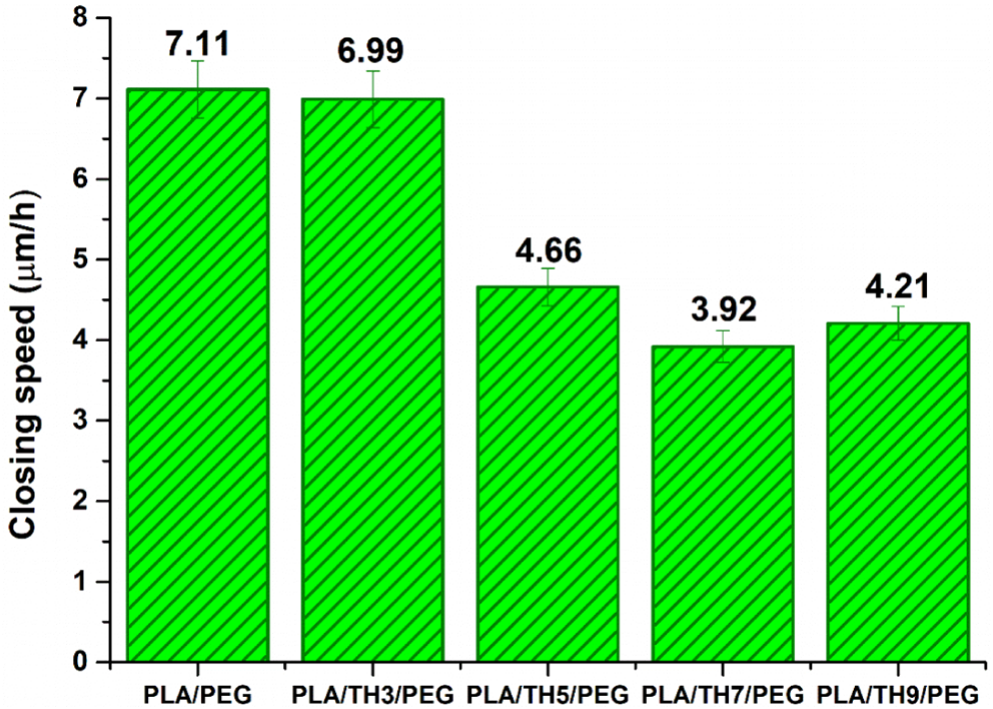
Time-dependent wound healing (μm/h) graphs of nanofibrous materials.

The amount of salt per unit area in the PLA/TH5/PEG nanofibrous material is less than that in PLA/TH9/PEG nanofibers. In this way, proliferating cells can find a passageway. However, in the presence of 9 wt % TH salt, it was predicted that there were enough fibroblasts in the environment to close the wound, although the passageway of cells decreased. Hence, it was stated that PLA/TH5/PEG and PLA/TH9/PEG nanofibrous materials exhibited a wound-closing speed close to each other. It was thought that the amount of salt per unit area in the PLA/TH7/PEG nanofiber both prevented fibroblast migration and did not provide as intense cell growth as supported by the 9 wt % TH salt additive. For this reason, the PLA/TH7/PEG nanofibrous material showed the lowest wound-closing speed with a value of 3.92 μm/h.

While L929 cell viability increased with TH incorporation (up to ∼122.7% at 9 wt %), the wound closure rate decreased, particularly at higher TH contents, suggesting that proliferation and migration are regulated by different biological mechanisms. The WST-1 assay primarily reflects cellular metabolic activity and proliferation potential, whereas the wound healing assay mainly evaluates collective cell migration.

### Antibacterial Activity Test of TH-Loaded PLA/PEG Nanofibrous Materials

The antibacterial activity of pure PLA/PEG and PLA/TH/PEG nanofibers against *E. coli* and *S. aureus* bacteria at the end of 24 h is shown in Figure 8 on the plates. The consistency and reliability of the test were ensured with the bacterial control plate. Pure PLA/PEG nanofibers have no antibacterial activity. Pure PLA/PEG nanofibrous material was used as a control sample to evaluate the antibacterial activity of the salt-added nonwoven nanofibers. It was observed that the obtained PLA/TH/PEG nanofibrous materials did not have antibacterial activity against *E. coli* or *S. aureus* cultures. It was also noted that innumerable bacteria grew on the plates at the end of 24 h. This may be attributed to the molecular structure and limited surface migration within the polymer matrix. The bulky tetrabutylammonium cation and the hexafluorophosphate counterion may reduce water solubility and restrict effective interaction with bacterial membranes. Therefore, although TH contributes to morphological and thermal modifications, its antimicrobial performance is limited under the tested conditions. As a result, it was reported that the changing addition rates of TH salt had no effect on the antibacterial activity behavior.^51^

**Figure 8:**
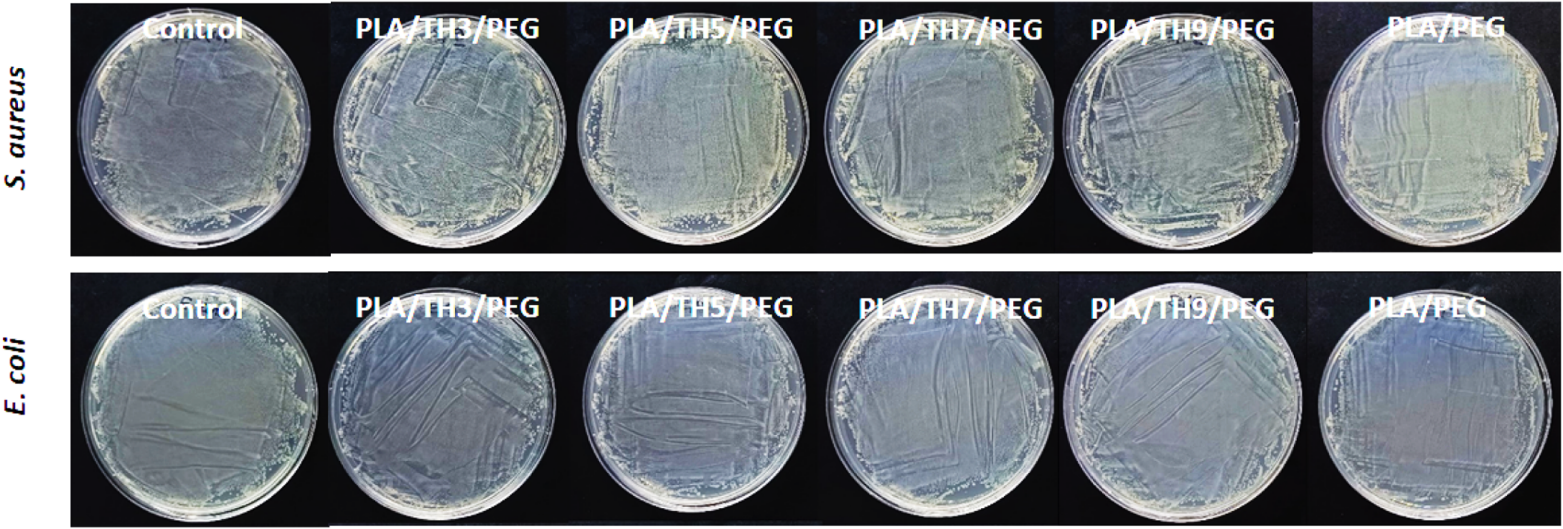
Plate images of nanofibrous materials against *E. coli*and *S. aureus* bacteria after 24 h.

## Conclusion

In this study, nanofibers were produced by electrospinning by adding different ratios of the TH salt to the PLA/PEG matrix. Comprehensive characterization and biological evaluation of the nanofibrous materials were conducted. According to the SEM results of the nanofibrous materials, the beaded structure observed in the PLA/PEG mat was eliminated upon the addition of the TH salt. Among the nanofibrous materials containing TH, the nanofibrous material with 9 wt % salt exhibited the most homogeneous and smooth morphology, as well as the smallest fiber diameter. In the LAC analysis of nanofibrous materials, the PLA/TH5/PEG nanofiber demonstrated the highest liquid absorption capacity (555.6%). In terms of liquid absorption capacity, the TH salt saturation point in the PLA/PEG matrix was evaluated to be 5 wt %. Moreover, it was reported that PLA/TH/PEG nanofibrous materials did not exhibit antibacterial activity against *E. coli* or *S. aureus* cultures. According to the wound healing test, the wound healing rate of PLA/TH/PEG nanofibers decreased with increasing TH content. However, the cell viability of the nanofibrous materials to which TH salt was added increased by approximately 21–31% compared to pure PLA/PEG nanofibers. This result indicates that TH salt acts as a nutrient for fibroblast cells and is noncytotoxic under the in vitro conditions tested. It is proposed that the produced PLA/TH/PEG nanofibers can be used as potential dermal wound dressings.

In future studies, cell viability analyses over longer durations (e.g., 48 and 72 h) could be performed to more comprehensively evaluate time-dependent cytotoxicity, and water vapor transmission rate (WVTR) analyses could be conducted to validate porosity results and further investigate nanofiber permeability.
